# A Comparison of Responses to Raised Extracellular Potassium and Endothelium-Derived Hyperpolarizing Factor (EDHF) in Rat Pressurised Mesenteric Arteries

**DOI:** 10.1371/journal.pone.0111977

**Published:** 2014-11-05

**Authors:** Alastair M. Mathewson, William R. Dunn

**Affiliations:** Pharmacology Research Group, School of Life Sciences, University of Nottingham, Nottingham, United Kingdom; The Chinese University of Hong Kong, Hong Kong

## Abstract

The present study examined the hypothesis that potassium ions act as an endothelium-derived hyperpolarizing factor (EDHF) released in response to ACh in small mesenteric arteries displaying myogenic tone. Small mesenteric arteries isolated from rats were set up in a pressure myograph at either 60 or 90 mmHg. After developing myogenic tone, responses to raising extracellular potassium were compared to those obtained with ACh (in the presence of nitric oxide synthase and cyclo-oxygenase inhibitors). The effects of barium and oubain, or capsaicin, on responses to raised extracellular potassium or ACh were also determined. The effects of raised extracellular potassium levels and ACh on membrane potential, were measured using sharp microelectrodes in pressurised arteries. Rat small mesenteric arteries developed myogenic tone when pressurised. On the background of vascular tone set by a physiological stimulus (i.e pressure), ACh fully dilated the small arteries in a concentration-dependent manner. This response was relatively insensitive to the combination of barium and ouabain, and insensitive to capsaicin. Raising extracellular potassium produced a more inconsistent and modest vasodilator response in pressurised small mesenteric arteries. Responses to raising extracellular potassium were sensitive to capsaicin, and the combination of barium and ouabain. ACh caused a substantial hyperpolarisation in pressurized arteries, while raising extracellular potassium did not. These data indicate that K^+^ is not the EDHF released in response to ACh in myogenically active rat mesenteric small arteries. Since the hyperpolarization produced by ACh was sensitive to carbenoxolone, gap junctions are the likely mediator of EDH responses under physiological conditions.

## Introduction

A number of factors are released from the vascular endothelium that act to modify vascular smooth muscle tone, including some which cause endothelium-derived hyperpolarisation (EDH). The identity of the factors causing EDH remains unclear with potential candidates including potassium ions [1], hydrogen peroxide, [2], [3] epoxyeicosatrienoic acids [4] or the passive transfer of charge/molecules through intercellular gap junctions [5].

Edwards *et al* (1998) reported that both raised extracellular potassium and EDHF produced vascular smooth muscle hyperpolarisation and vasorelaxation in rat hepatic and mesenteric arteries [1]. The hyperpolarising and vasorelaxant responses to both potassium and EDH were abolished by the combination of inhibitors of Na^+^/K^+^ ATPase and inwardly rectifying potassium channels (K_ir_). This observation, coupled with the detection of potassium released from the endothelium in response to acetylcholine (ACh), led to the suggestion that potassium was an EDHF in these arteries.

Subsequent studies questioned the role of potassium as an EDHF in rat mesenteric small arteries, largely on the basis of inconsistencies observed in the functional vasorelaxant responses produced by raising extracellular potassium in comparison to the EDHF released in response to ACh [6]–[8]. To account for these disparate findings, it was proposed that the experimental methodology employed in the latter studies minimised the potential for establishing an important role for potassium as an EDHF. Most studies examining vasodilator responses to raised extracellular potassium have induced tone with an alpha_1_-adrenoceptor agonist, such as phenylephrine. It has been shown that the depolarisation associated with phenylephrine-induced contractile responses evokes the release of potassium from vascular smooth muscle cells, via calcium-activated potassium channels (K_Ca_). This leads to the accumulation of a “potassium cloud” around vascular myocytes, which increases background activation of Na^+^/K^+^ ATPase, thus reducing the scope for potassium-induced hyperpolarisation and vasorelaxation [9], [10]. These observations led to the proposal that the presence of potassium clouds in vasospastic arteries would substantially reduce the scope for potassium acting as an EDHF, but that with more moderate levels of smooth muscle activation potassium could have an important physiological role as an EDHF [10].

Small arteries develop myogenic tone in response to raised intraluminal pressure, an effect which becomes more pronounced as the size of the vessel decreases [11], [12]. Pressure-induced myogenic tone sets the physiological background level of vasoconstriction against which vasodilators, such as EDHF, produce their effects [13]. Therefore, in the present study, we compared vasodilator and electrophysiological responses to raised extracellular potassium with the EDHF released by ACh, in myogenically active mesenteric small arteries isolated from rats. The data indicates that potassium ions are not the EDHF released under physiological conditions.

## Materials and Methods

Male Wistar rats (150–200 g) were killed by stunning and exsanguination, using an approved Schedule 1 method of euthenasia. All procedures were approved by the animal welfare and ethical review body of the University of Nottingham. The gastrointestinal tract, with the mesenteric arcade attached, was excised and placed in physiological salt solution (PSS) at 4°C. Third and fourth order arteries were dissected clean of any connective tissue and secured between two glass cannulae of a pressure myograph (Living Systems Instrumentation, Burlington, VT, USA) [14], [15]. One cannula was attached to a pressure-servo system containing PSS, allowing the control of intraluminal pressure and measurement of diameter changes under isobaric conditions. After residual blood was flushed from the lumen, the other cannula was sealed and the vessel checked for leaks. Arteries were superfused with PSS gassed with 5% CO_2_:95% O_2_ and the temperature of the organ bath was maintained at 36°C. The vessels were imaged using a video camera and the diameter was measured using a dimension analyser (Living Systems Instrumentation), linked to a MacLab data acquisition system. All experiments were performed in the presence of indomethacin (1 µM) and nitro-L-arginine methyl ester (L-NAME; 100 µM) to block cyclo-oxygenase and nitric oxide synthase respectively.

### Pressure myography

Intraluminal pressure was set at 60 or 90 mmHg and vessels were allowed to equilibrate for 60 min during which time they developed a variable degree of myogenic tone. Vessels which did not reduce their diameter by more than 10% were excluded from further experimentation. Following the development of stable myogenic tone, cumulative concentration response curves to either exogenous potassium (+1 to +15 mM), additional to the potassium concentration already present, (i.e. 6 mM) or ACh (10 nM to 10 µM) were constructed by adding them to the external superfusing solution. In some experiments, the responses to potassium or ACh were assessed after 30 min exposure to the combination of Barium (Ba^2+^, 30 µM) and ouabain (1 µM or 1 mM). The role of sensory nerves in mediating the vasodilator response to potassium and ACh was also assessed. Sensory neurotransmitter depletion was achieved by treatment with capsaicin (1 µM) for 40 min followed by a thirty minute washout period. The responses to either potassium or ACh were re- assessed once the artery had developed stable myogenic tone.

### Electrophysiology

Rat mesenteric arteries were mounted in a pressure myograph system as described above. It proved impossible to maintain cellular impalements in myogenically active arteries when they were exposed to vasodilators, due to vessel movement. Therefore, electrophysiological responses to potassium or ACh were conducted in two ways; at a sub-myogenic pressure (30 mmHg) or, at 90 mmHg in the presence of the non-selective myosin light-chain kinase inhibitor, wortmannin (1 µM), which abolished the myogenic response, without interfering with the underlying membrane potential [16], [17]. Intracellular recordings were made from smooth muscle cells located near the adventitial-medial border of the mesenteric artery with borosilicate glass microelectrodes (80–160 MΩ) filled with 0.5 M KCl and connected to an Axoclamp bridge amplifier (Axon Instruments Inc., Foster City, USA). The criteria for accepting impalements, were the same as those adopted by Brock and Van Helden [18] with resting membrane potential determined upon withdrawal of the microelectrode. Changes in membrane potential were measured in response to single concentrations of ACh (1 µM) and potassium (+5 mM), applied in a random order. Some experiments examined the effect of carbenoxolone (100 µM) on responses to ACh (1 µM) at 30 mmHg.

### Drugs and solutions

The composition of PSS was as follows (mM): NaCl 118, NaHCO_3_ 25, glucose 11.1, KCl 4.8, MgSO_4_ 2.5, KH_2_PO_4_ 1.2 and CaCl_2_ 1.25. Ca^2+^-free PSS composition was as above with the addition of EGTA (0.5 mM) and the omission of CaCl_2_. Responses to potassium were obtained by adding high-potassium PSS to normal PSS to obtain the desired final concentration. High-potassium (45 mM) PSS was prepared by equimolar replacement of NaCl with KCl. Acetylcholine, barium chloride, carbenoxolone, ouabain, wortmannin, capsaicin, L-NAME and indomethacin were obtained from Sigma, UK. All drugs were freshly prepared in PSS from stock solutions on the day of use. Indomethacin, capsaicin and wortmannin were prepared in ethanol and ouabain was prepared in 20% ethanol in distilled water (v/v). The final bath concentration of ethanol did not exceed 0.1% (v/v) and did not affect vascular function.

### Data analysis

Myogenic tone was calculated as the % reduction in vessel diameter measured at the start of the experiment and after the development of stable myogenic tone. Vasodilator responses to potassium and ACh were expressed as percentages of the maximum possible vasodilator range i.e. the difference in vessel diameter in Ca^2+^-free PSS and after the development of myogenic tone. Results are presented as mean ± s.e.mean with *n* = to the number of rats. R_max_ refers to the maximum vasodilator response obtained during the construction of a cumulative concentration response curve to either potassium or ACh. Statistical analysis was performed using GraphPad Prism. EC_50_ values were calculated for individual concentration response curves using nonlinear regression (sigmoidal dose-response). Differences in maximum dilator response (R_max_) and smooth muscle cell hyperpolarisation were compared with an unpaired or paired Student’s t-test respectively. A *P*<0.05 was considered statistically significant. Differences between the potassium or ACh concentration-response curves, before and after Ba^2+^ and ouabain treatment, were assessed using a repeated measures two-way analysis of variance (ANOVA).

## Results

### Pressure myography

In the presence of L-NAME and indomethacin, third and fourth order rat mesenteric arteries (diameter: 216±5 µm, n = 49) spontaneously reduced their diameter in response to an increase in intraluminal pressure. At 60 mmHg, 31% (8/26) of vessels tested spontaneously reduced their diameter in response to the pressure stimulus with a mean reduction in diameter of 18±3% (*n* = 8). A greater proportion of the vessels pressurised to 90 mmHg, developed tone, 75% (41/55). Moreover, the size of the reduction in diameter was greater; 35±2% (*P*<0.05). Arteries which developed less that 10% myogenic tone were excluded from further study.

**Figure 1:**
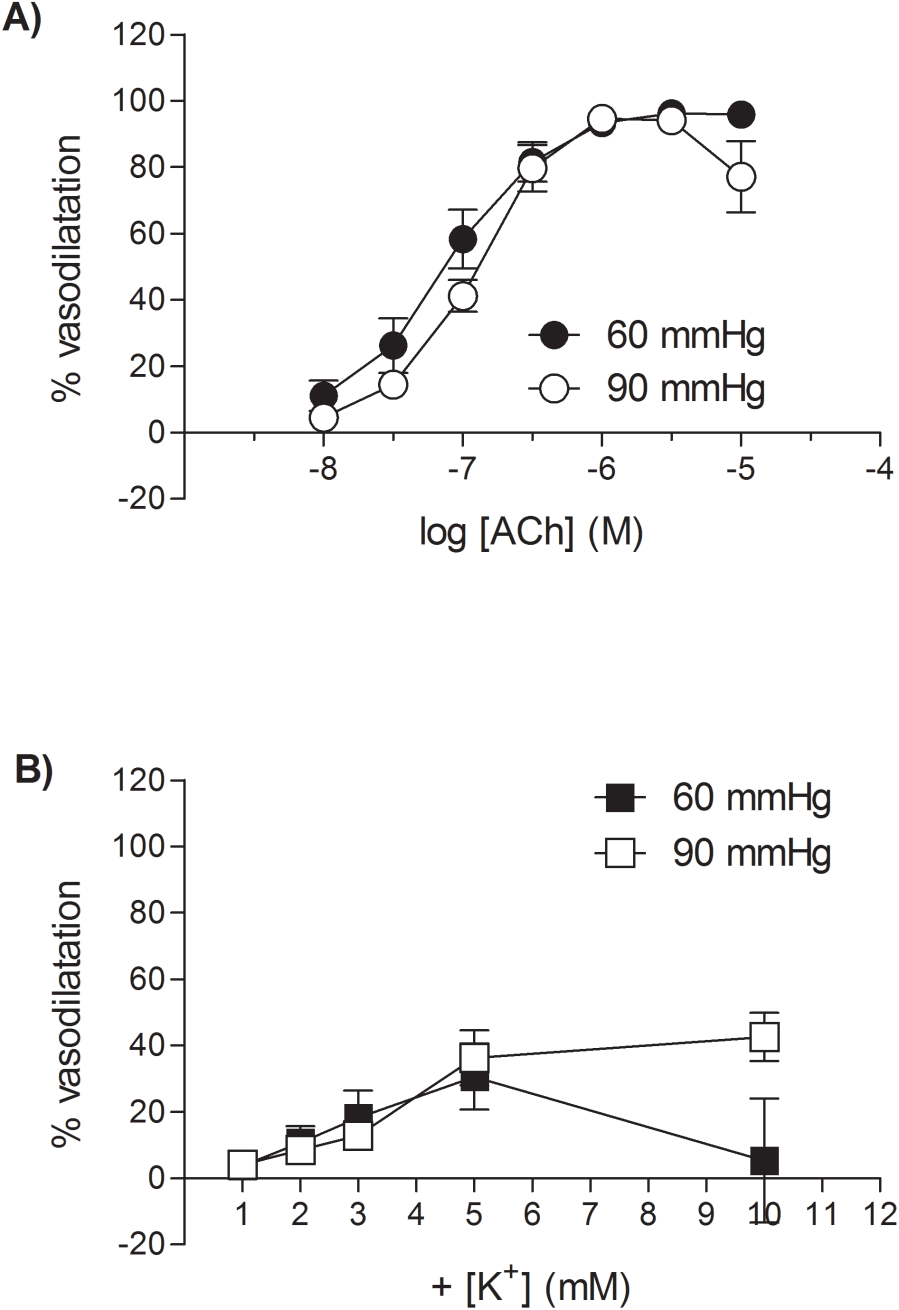
ACh produces a larger vasodilator response than raising extracellular potassium in myogenically-active mesenteric small arteries. Vasodilator responses to (A) ACh and (B) raised extracellular potassium in rat isolated mesenteric small arteries pressurised to 60 mmHg or 90 mmHg (in the presence of L-NAME and indomethacin). Following the development of stable myogenic tone, cumulative concentration response curves were constructed to ACh or potassium in random order in the same artery from *n* different animals. Each point represents the mean ± s.e.mean (n = 6–9).

ACh, in the presence of combined inhibition of cyclo-oxygenase and nitric oxide synthase, produced concentration-dependent vasodilator responses in vessels displaying myogenic tone at both 60 and 90 mmHg (figure 1A), with a similar maximum response (R_max_: 97±2%, *n* = 7 (60 mmHg); 95±1%, *n* = 9 (90 mmHg)) and sensitivity (log EC_50_: −7.23±0.14; *n* = 7 (60 mmHg); −7.09±0.07; *n* = 9 (90 mmHg)). The vasodilator responses to raising extracellular potassium in mesenteric small arteries (figure 1B), despite being variable (R_max_: −1 to 96% maximum diameter), did not differ significantly when measured at either 60 or 90 mmHg (60 mmHg: 35±9% maximum relaxation; *n* = 6; compared to 90 mmHg: 49±9% maximum relaxation; *n* = 9). When comparing responses to ACh and potassium in the same vessel, vasodilator responses to ACh were greater in magnitude (range 60 mmHg: 92–100% maximum diameter and 90 mmHg: 82–100% maximum diameter) than responses to potassium (range 60 mmHg: −1–53% maximum diameter and 90 mmHg: 12–96% maximum diameter), regardless of the order in which they were applied.

**Figure 2:**
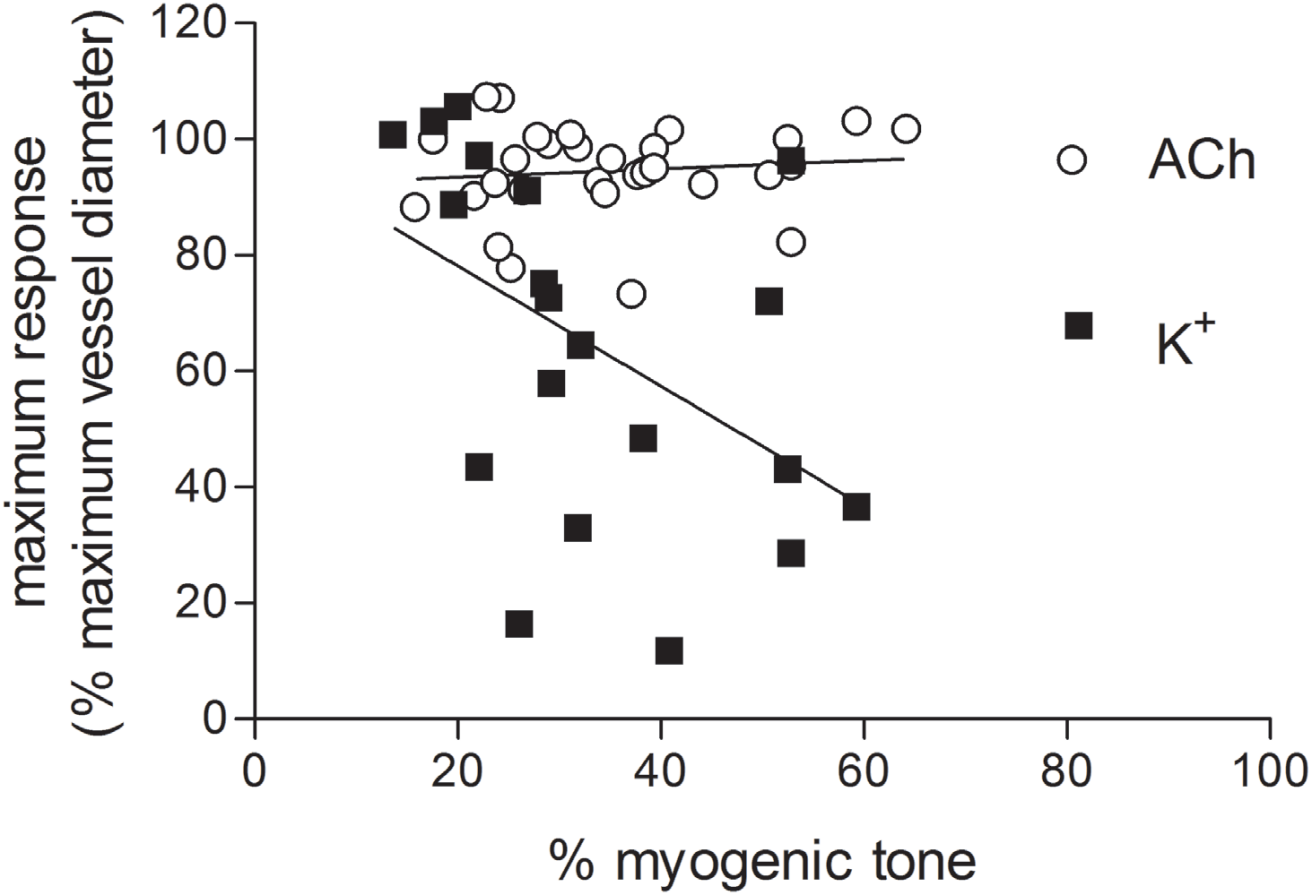
Vasodilator responses to raised extracellular potassium correlate with the degree of myogenic tone. The relationship between the level of myogenic tone (in the presence of L-NAME (100 µM) and indomethacin (1 µM)) and the R_max_ for ACh (*n* = 31) or raised extracellular potassium (*n* = 20) in rat isolated mesenteric arteries pressurised to 90 mmHg. Each point represents one experiment. The slope of the line fitted to the potassium data was significantly different from zero (*P*<0.05) with an r^2^ = 0.2387. The slope of the line fitted to the ACh data was not significantly different from zero.

The maximum vasodilator response to potassium at 90 mmHg showed a significant correlation with the level of prevailing myogenic tone (figure 2), whereby the magnitude of potassium responses decreased as the level of myogenic tone increased (*P*<0.05, r^2^ = 0.2387, *n* = 20). Responses to ACh did not vary with the degree of myogenic tone (figure 2).

**Figure 3:**
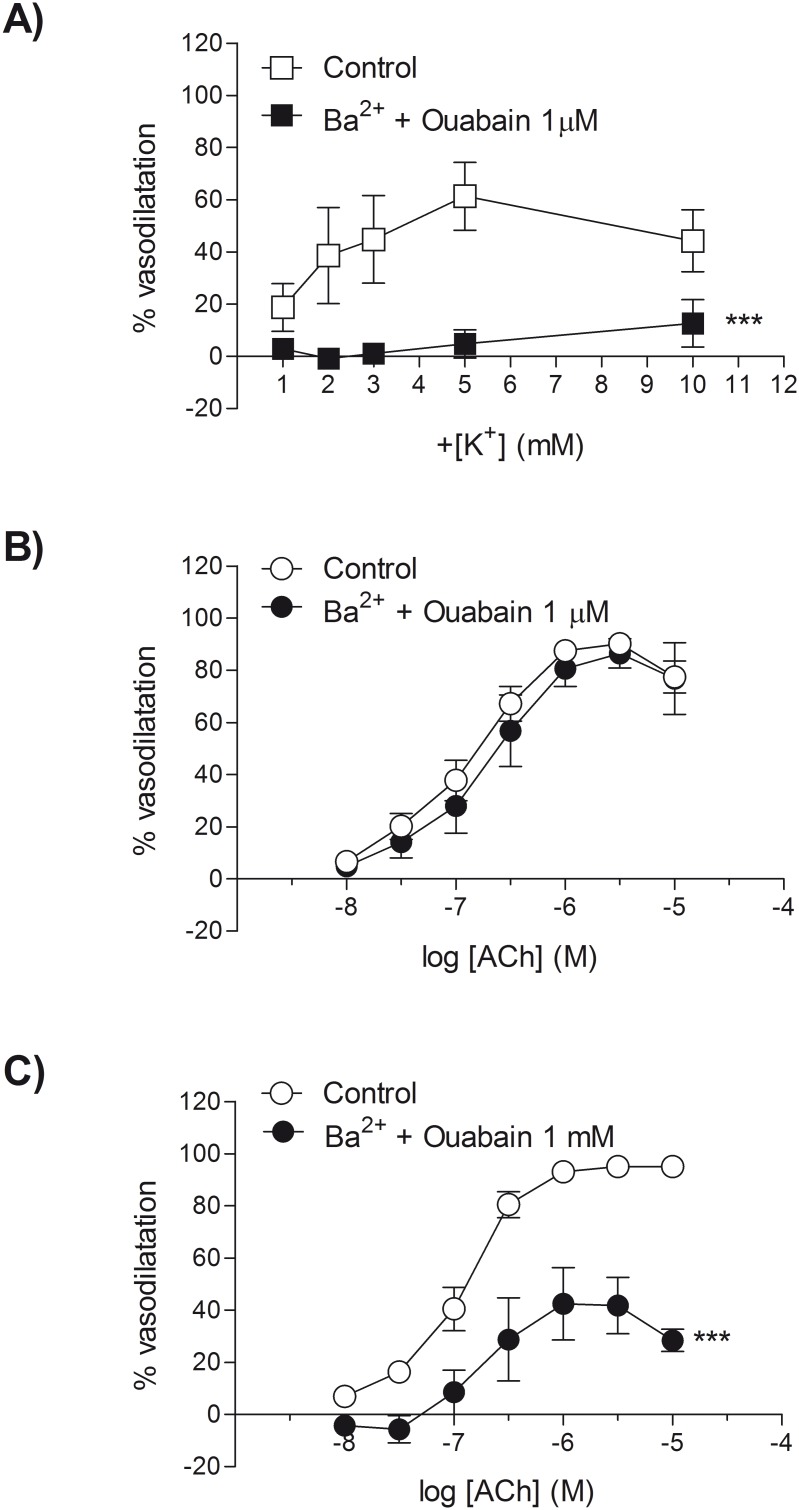
Responses to raised extracellular potassium are sensitive to the combination of barium and ouabain. The effect of Ba^2+^ (30 µM) and ouabain (1 µM or 1 mM) on responses to raised extracellular potassium (A) in rat isolated mesenteric small arteries at 90 mmHg. Responses to ACh in the presence or absence of Ba^2+^ (30 µM) and ouabain (B: 1 µM or C: 1 mM). All experiments were carried out presence of L-NAME (100 µM and indomethacin (1 µM). Each point represents the mean ± s.e.mean. ***P<0.001, Ba^2+^ and ouabain treated versus control, *n* = 5.

A proposed mechanism for the vasodilator response produced by potassium is through activation of Na^+^/K^+^ ATPase and K_ir_ channels with a resultant hyperpolarisation. Incubation with ouabain (1 µM) and Ba^2+^ (30 µM) abolished the vasodilator response to potassium (figure 3A). The maximum vasodilator response to exogenous potassium was significantly reduced following ouabain and Ba^2+^ treatment (R_max_ 16.6±8.3% relaxation versus control 73.3±10.3% relaxation; *P*<0.05; *n* = 5). In contrast, treatment with ouabain (1 µM) and Ba^2+^ (30 µM) did not affect the vasodilator response to ACh (R_max_ 89.2±6.0% relaxation versus control 90.6±2.8% relaxation; *n* = 5) (figure 3B). By increasing the concentration of ouabain by 1000-fold to 1 mM, and in the presence of Ba^2+^ (30 µM), vasodilator responses to ACh were significantly reduced (R_max_ 46.8±12.9% relaxation versus control 95.5±1.3% relaxation; *P*<0.05; *n* = 5) but not abolished (figure 3C). The incubation with Ba^2+^ and ouabain (1 µM and 1 mM) did not significantly affect the level of prevailing myogenic tone (potassium experiments (1 µM ouabain and 30 µM Ba^2+^): before 22±3% myogenic tone; after 33±6% myogenic tone. ACh experiments (1 µM ouabain and 30 µM Ba^2+^) before: 27±2% myogenic tone; after 22±3% myogenic tone. ACh (1 mM ouabain and 30 µM Ba^2+^) before: 34±1% myogenic tone; after 32±3% myogenic tone. *n* = 15).

**Figure 4:**
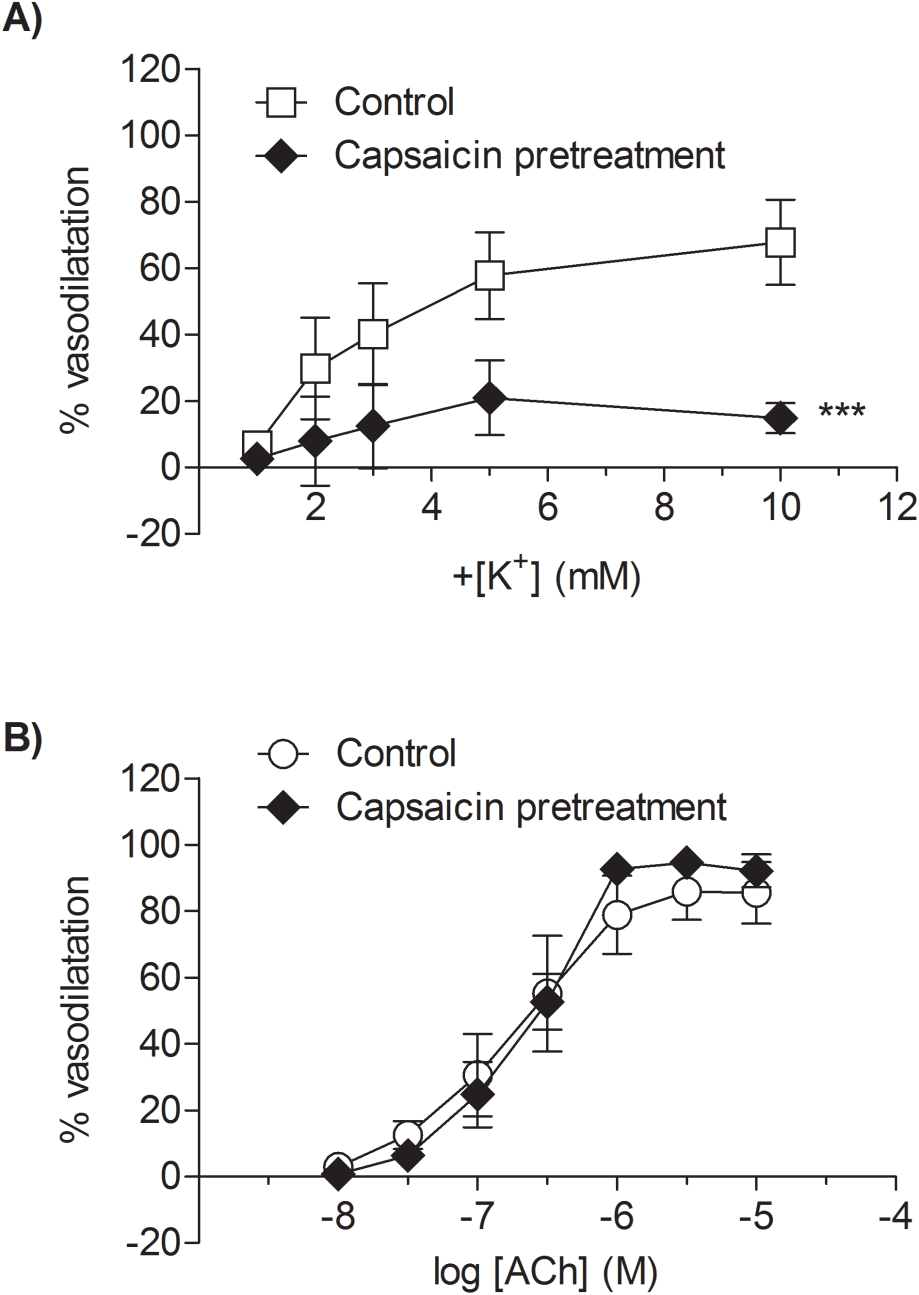
Responses to raised extracellular potassium, but not ACh, are capsaicin sensitive. The effect of capsaicin on responses to raised extracellular potassium (A) or ACh (B) in rat isolated mesenteric small arteries at 90 mmHg. All experiments were carried out presence of L-NAME (100 µM and indomethacin (1 µM). Each point represents the mean ± s.e.mean. ***P<0.001 capsaicin versus control; *n* = 4–5.

Following sensory nerve depletion with capsaicin (1 µM) [19], the concentration-dependent vasodilator response to potassium was significantly inhibited (*P*<0.01; *n* = 5; Fig. 4A). In contrast, the vasodilator response to ACh was unaffected by pre-treatment with capsaicin (Fig 4B). The maximum vasodilator response to potassium was significantly reduced following capsaicin treatment (R_max_: 26.2±10.3% relaxation versus control 69.5±12.7%; *P*<0.05; *n* = 5) whereas the maximum vasodilator response to ACh was unaffected (R_max_: 95.3±2.4% relaxation versus control: 86.2±8.7% relaxation; *n* = 4).

### Electrophysiology

**Figure 5:**
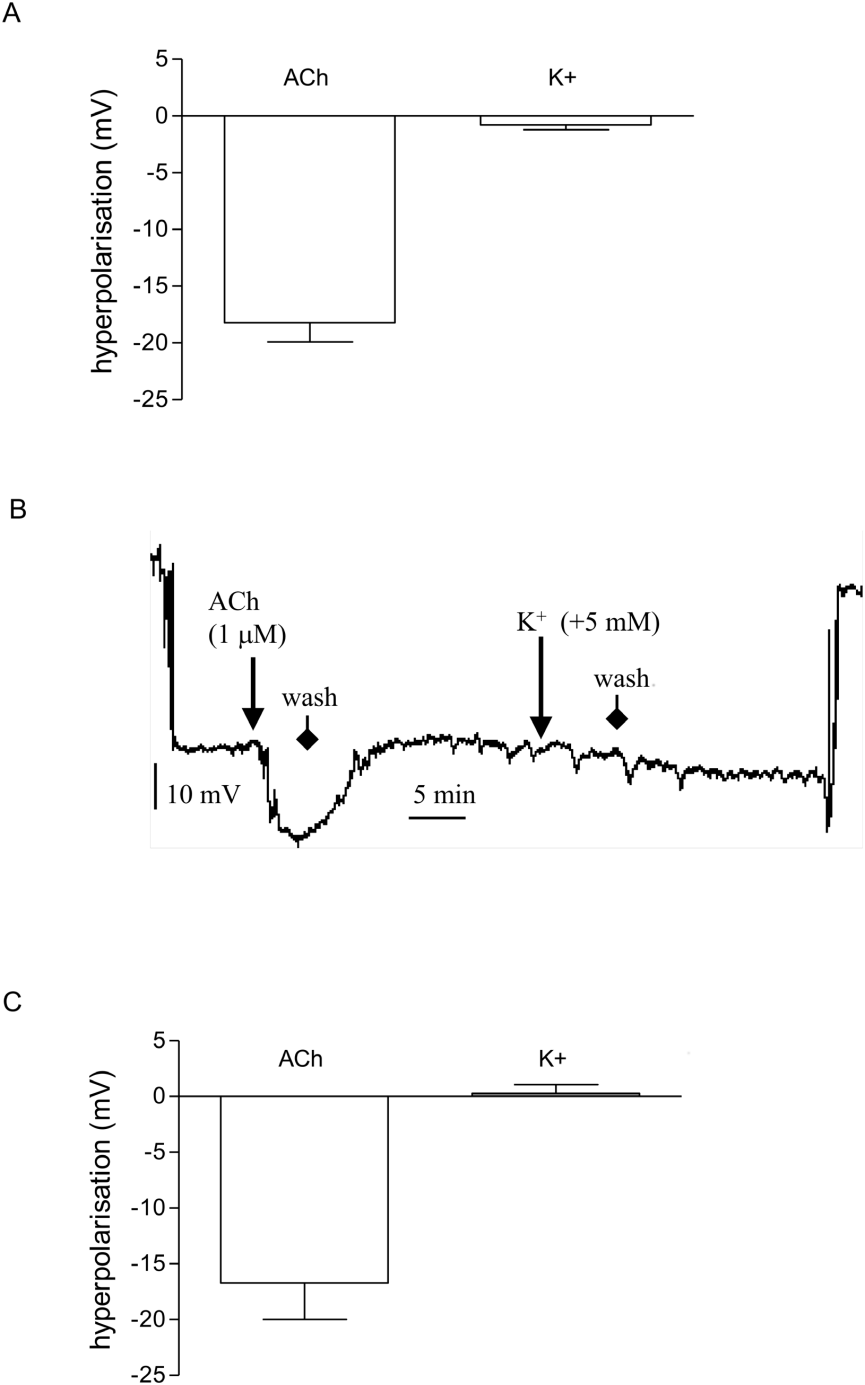
ACh causes hyperpolarization in pressurized small arteries, while raising extracellular potassium does not. Summary data showing the change in membrane potential in smooth muscle cells from *n* different animals in response to ACh (1 µM) and raised extracellular potassium (+5 mM) at (A) 30 mmHg and (C) 90 mmHg. Following the successful impalement of a cell, responses to ACh or potassium were recorded in random order. Bars represent mean ± s.e.mean (*n* = 3–6). Representative trace recording (B) of the change in membrane potential of a rat mesenteric small artery held at 30 mmHg and exposed to ACh (1 µM) followed by raised extracellular potassium (+5 mM). The large deflections at the start and end of the trace recording reflect cell impalement and removal of the microelectrode, respectively.

In electrophysiological studies, ACh (1 µM) produced a substantial hyperpolarisation of the vascular smooth muscle (–18.2±1.7 mV, *n* = 6) whereas no response to raising extracellular potassium (+5 mM) was observed (–0.8±0.4 mV, *n* = 6; figure 5A) in vessels held at 30 mmHg, a pressure below the myogenic threshold. The resting membrane potential of arteries held at 30 mmHg was –46.0±1.0 mV (*n* = 6). A representative trace of the effects of ACh and potassium in a single cell is shown in figure 5B. Due to the induced vasodilator response, it was impossible to record changes in membrane potential in myogenically-active vessels. Therefore, some arteries, held at 90 mmHg, were exposed to wortmannin (1 µM), which abolished tone. The resting membrane potential of vessels under these conditions was significantly more positive, −34.9±3.8 mV (*n* = 5), than at 30 mmHg (*P*<0.05). At 90 mmHg, ACh (1 µM) produced a hyperpolarisation of the vascular smooth muscle (–16.7±3.3 mV, *n* = 5), of similar magnitude to that produced in vessels held at 30 mmHg, while potassium had virtually no effect (0.3±0.8 mV, *n* = 3; figure 5C). Carbenoxolone (100 µM) attenuated responses to Ach (1 µM) in arteries held at 30 mmHg (control; −16.5±3.9 mV: carbenoxolone −7.7±2.4 mV *n* = 4; P<0.05 Student’s t-test).

## Discussion

The results from the present study indicate that potassium ions do not act as the EDHF released in response to ACh in pressurised mesenteric small arteries displaying myogenic tone. Vasodilator responses to ACh were invariably larger than responses to potassium in small arteries; responses to ACh displayed relative resistance to inhibition by the combination of barium and oubain, or capsaicin, in contrast to potassium -induced vasodilator responses; most importantly, ACh caused vascular smooth muscle hyperpolarisation in pressurised blood vessels while raising extracellular potassium did not.

In 1998, Edwards and colleagues demonstrated, by electrophysiological means, that EDHF-mediated responses in rat mesenteric and hepatic arteries were mimicked by raising extracellular potassium [1]. However, several functional studies failed to provide support for these observations [6]–[8], [20], [21]. A reason proposed for this discrepancy was that the use of contractile agents in the functional experiments was excessive and provided a background level of stimulation which impaired vasorelaxant responses to potassium [22], [23]. Indeed, the proposition of a vasoconstrictor-induced potassium cloud has led to the suggestion that the majority of studies using a pre-constrictor agent mimic vasospastic conditions, thereby minimising the physiological importance of potassium as an EDHF [9], [10], [23]. The present study showed marked differences in responses to EDHF and potassium in small mesenteric arteries isolated from rats displaying the most pertinent physiological determinant of small artery vasoconstriction, namely, myogenic tone, suggesting that potassium ions do not act as a physiological EDHF in this vascular bed.

When small arteries are pressurised they frequently develop myogenic tone associated with vascular smooth muscle depolarization [24], [25]. While the pressure-induced myogenic response is easily demonstrated in small arteries isolated from certain vascular beds (e.g. cerebral vessels), the presence of pressure-induced myogenic tone in small mesenteric arteries is more variable. Some studies have shown that mesenteric arteries develop myogenic tone [11], [12], [26] while others have not [7], [27]. The reasons for this may reflect the size of vessels studied, the pressure under which they are held or other factors related to experimental protocols. The arteries used in the present study had a mean, pressurised diameter of 216±5 µm. Pressure-induced myogenic tone was evident in approximately 30% of vessels held at 60 mmHg and in 75% of vessels held at 90 mmHg, while at 30 mmHg vessels did not display an active vasoconstriction. The inhibition of NO release by L-NAME may have increased the magnitude of the pressure-induced myogenic tone in the present study. It is difficult to estimate the pressure arteries of this size would experience *in* *vivo*. However, the best estimates obtained from conscious rats suggest values in the region of 75% of the mean arterial pressure, which translates into a pressure in the range of 60–90 mmHg [28], [29]. Thus, the data generated in the present study can be considered in the physiological pressure range.

Under physiologically relevant levels of smooth muscle activation, the vasodilator responses to raised extracellular potassium were inconsistent, ranging from almost no response to a maximum possible vasodilatation. The size of the vasodilator response to potassium was negatively correlated with the degree of myogenic tone. The magnitude of response to potassium has previously been shown to be influenced by the degree of vascular smooth muscle activation produced by vasoconstrictor agents, such as phenylephrine [9], [22], [23], [27]. The model proposed to explain these findings suggests that activation of myocytes leads to the efflux of potassium ions from smooth muscle cells via K_Ca_ channels. This potassium cloud is reputed to maximally activate K_ir_ and Na^+^/K^+^ATPase, whereby the addition of more extracellular potassium can no longer produce a vasodilator response. This has been shown both in isometrically-mounted arteries and in pressurised vessels, where vasodilator responses to potassium were only evident after pre-treatment with K_Ca_ blockers in the presence of phenylephrine-induced vasoconstriction [9], [23], [27]. In one of the latter studies pressurised mesenteric arteries did not develop spontaneous myogenic tone, perhaps because they were larger (>300 µm) than the vessels used in the present study [27]. While, our observations could be taken as support for the hypothesis proposed by Dora and Garland [22] and Edwards and Weston [10] that, as smooth muscle activation increases so the scope for potassium-induced vasodilatation decreases, we have shown that raised extracellular potassium causes the release of sensory neuropeptides in myogenically active arteries (see below) and thus, higher levels of tone may just as easily impact on the extent of vasodilatation produced by the release of sensory neuropeptides. In contrast to the responses produced by raised extracellular potassium, ACh produced consistent vasodilator responses that were always large in magnitude, even in those preparations in which the response to potassium was small or absent, and these responses did not change in relation to the extent of smooth muscle activation. Thus the EDHF released by ACh is disparate from potassium in vessels displaying physiologically relevant myogenic tone.

Further separation between the responses produced by potassium and the EDHF released by ACh in myogenically active arteries, was shown by their sensitivity to Na^+^/K^+^ ATPase and K_ir_ channel inhibition with ouabain (1 µM) and Ba^2+^ (30 µM), respectively. Na^+^/K^+^ ATPase and K_ir_ channels were involved in mediating both EDHF and potassium-induced hyperpolarisations in rat mesenteric and hepatic arteries [1]. This combination of inhibitors abolished the vasodilator responses to potassium but was without effect on responses to ACh. Myogenic tone was unaffected by Na^+^/K^+^ ATPase and K_ir_ channel inhibition and therefore differences in sensitivities to Ba^2+^ and ouabain cannot be explained by alterations in smooth muscle activation. The sensitivity of the ACh responses to the combination of Ba^2+^ and ouabain has been reported elsewhere [8], [9]. However, in this study the responses to ACh were only sensitive to ouabain after a 1000-fold increase in the concentration to 1 mM, a concentration that has been used previously to block EDHF [1]. Furthermore, high concentrations of ouabain (1 mM) can impair gap junction communication [30], [31], which may offer an explanation for the inhibition of the ACh-mediated EDHF response. Unfortunately, the gap junction inhibitors carbenoxolone and 18-α glycyrrhetinic acid abolished the myogenic response in these arteries, an effect also observed by others [26], [32], and thus prevented us from determining whether gap junctions were involved in mediating the vasodilator responses to ACh. However, carbenoxolone attenuated the electrophysiological response to ACh in arteries held at 30 mmHg indicating that gap junctions are the likely mechanism of EDH under pressurized conditions. These data support previous observations made in rat mesenteric arteries, which have shown a role for gap junctions in mediating EDH responses, after inducing tone with α-adrenoceptor agonists [33], with CX40 being crucially important [34].

Vasodilator responses to raised potassium in rat mesenteric small arteries were sensitive to depletion of sensory neurotransmitters using capsaicin. Capsaicin pre-treatment has been shown to attenuate potassium-induced vasodilator responses in the rat hepatic artery [35], [36] and to prevent potassium-induced increases in tooth pulp blood flow [37]. In addition, Zygmunt *et al* [36] showed that ouabain pre-treatment was associated with the slow release of sensory neuropeptides and subsequent neurotransmitter depletion in guinea-pig basilar arteries, presumably by inhibiting the Na^+^/K^+^ATPase on sensory nerves. We suggest therefore, that raised extracellular potassium causes the release of sensory neuropeptides to cause vasodilatation and that both capsaicin and ouabain inhibit this response by depleting sensory nerves of their neurotransmitters. This mechanism explains how potassium can induce a vasodilator response without an associated smooth muscle cell hyperpolarization (see below).

While there were differences in the vasodilator response produced by ACh-induced EDHF release and raising extracellular potassium, the most direct comparison of the potential for potassium to act as an EDHF in pressurised vessels was obtained using sharp microelectrodes to measure changes in membrane potential. We examined pressurised arteries under two conditions; at a pressure below the myogenic threshold (30 mmHg) and at high pressure (90 mmHg) in the presence of wortmannin, which acts to inhibit myosin light-chain kinase [16]. Under both experimental conditions, ACh produced a hyperpolarization of the underlying smooth muscle, while raising extracellular potassium (with a concentration that caused vasodilatation) was without effect. Thus potassium is not an EDHF in myogenically-active, rat pressurised mesenteric small arteries.

In conclusion, the present study has shown that potassium is not the EDHF released in response to ACh in pressurised, myogenically active small mesenteric arteries isolated from rats. This conclusion is based on the relative size of responses to ACh and potassium, the relative resistance of responses to ACh to the combined inhibition of Na^+^/K^+^ ATPase and K_ir_ channels or sensory nerve depletion and, most importantly, the lack of a hyperpolarising response to raising the extracellular concentration of potassium in pressurised arteries.
